# Endovascular treatment of aortic stump blow-out after extra-anatomical repair of aortoduodenal fistula: a case report and review of literature

**DOI:** 10.1186/s42155-020-00111-8

**Published:** 2020-04-13

**Authors:** E. Beijer, V. P. W. Scholtes, P. Moerbeek, H. M. E. Coveliers, R. J. Lely, A. W. J. Hoksbergen

**Affiliations:** 1 Department of Surgery, Amsterdam University Medical Centre, location VUmc, P.O. Box 7057, 1007 MB Amsterdam, The Netherlands; 2 Department of Surgery, General City Hospital, Aalst, Belgium; 3 Department of Radiology, Amsterdam University Medical Centre, location VUmc, Amsterdam, the Netherlands

**Keywords:** Aortoduodenal fistula, Aortoenteric fistula, Aortic stump, Aortic stump blow-out, Aortic stump rupture, Endovascular, Endovascular treatment, Endovascular repair, Amplatzer®, Amplatzer® Vascular Plug

## Abstract

**Background:**

An aortoduodenal fistula (ADF) is an unusual, but serious complication following surgical or endovascular aortic repair. The optimal treatment for ADF consists of removal of the infected graft with in situ or extra-anatomical repair and is associated with high mortality. Part of this mortality is caused by re-bleeding or aortic stump ruptures. Classical treatment of an aortic stump rupture involves immediate re-laparotomy, removal of infected tissue, aortic stump formation and reinforcement with soft tissue flaps.

However, this invasive treatment is often difficult to perform and the condition of the patient frequently requires a more rapid response. We describe a case in which an aortic stump rupture was treated endovascularly by using an Amplatzer® Vascular Plug, which successfully stopped the bleeding.

**Case presentation:**

This report describes a 67-year-old man who was presented with persistent duodenal leakage (due to secondary duodenal perforation) after resection and open in-situ repair of an infected aorto-bi-femoral prosthetic graft. An extra-anatomical reconstruction was performed with an axillo-bi-femoral bypass, followed by excision of the prosthesis, aortic stump formation, partial duodenal resection and duodenojejunal reconstruction. Twelve weeks later, sudden severe hematemesis with severe hemodynamic instability occurred. Computed tomography angiography showed extravasation of blood from the aortic stump into the duodenal loop. Endovascular treatment of the aortic stump blow-out with an Amplatzer® Vascular Plug was performed, which successfully stopped the bleeding and stabilized the patient. The duodenal fistula was treated conservatively. Three months later, the patient was discharged to a rehabilitation clinic in a good clinical condition. The patient was still alive after a follow-up of 4 years.

**Conclusions:**

Rapid treatment is requested in cases of aortic stump rupture. Re-laparotomy is practically never the most suitable solution and most of these aortic stump ruptures are fatal. Endovascular treatment could be a suitable alternative. Whether the endovascular treatment of aortic stump rupture is a definitive treatment or a bridge to surgery remains to be elucidated.

## Introduction

An aortoduodenal fistula (ADF) is a relatively uncommon, but serious complication following surgical or endovascular aortic repair (Batt et al. 2011; Bergqvist and Björck 2009; Deijen et al. 2016). Local infection, mechanical erosion and endotension play a role in the development of an ADF (Saratzis et al. 2008). We describe a case of a patient who was treated for an ADF with extra-anatomical bypass grafting, aortic stump formation and partial duodenal resection, which was complicated by an aortic stump bleeding 3 months later. The bleeding was successfully treated endovascularly with an Amplatzer® Vascular Plug (AVP) (Abbott Vascular, Santa Clara, California).

## Case report

A 67-year-old man was presented to our hospital with persistent duodenal leakage after resection and in-situ repair of an infected aortic bifurcation prosthesis. The patient’s medical history showed a right-sided hemicolectomy (2005), elective open aneurysm repair with an aorto-bi-iliac prosthesis (2006), and incisional hernia repair with a mesh (2007). Three weeks before admission, in April 2013, replacement of an infected aorto-bi-iliac prosthesis was performed. The infection was caused by a secondary ADF. The patient underwent open repair with an aorto-bi-femoral prosthetic graft, which was complicated by right ureteric damage which was treated with a local ureter-plasty. After renewed sepsis due to persistent duodenal leakage, the patient was referred to our hospital. An extra-anatomical reconstruction was performed with a right-sided 8-mm Dacron® axillo-bi-femoral bypass, removal of the recent aorto-bi-femoral prosthesis, aortic stump formation and an eight-centimeter resection of the duodenum with a duodenojejunal anastomosis. The aortic stump was infra-renally closed with double rowed Prolene® 3.0 sutures incorporating felt drenched in Rifampicin®.

**Fig. 1:**
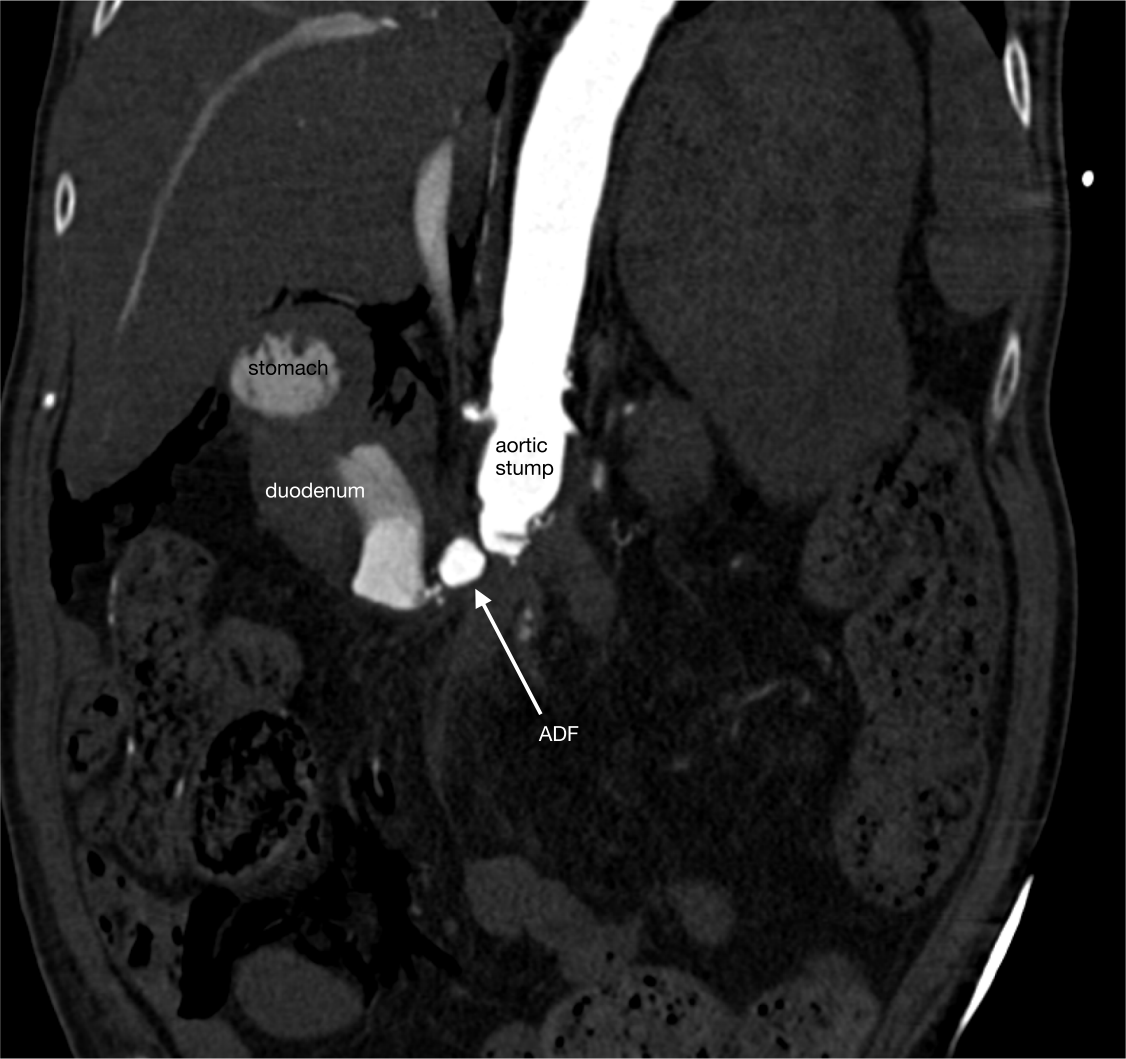
CTA abdomen showing massive extravasation of blood due to a secondary ADF CTA demonstrating massive extravasation of blood through the aortic stump into the duodenum, due to a secondary ADF (marked with an arrow).

**Fig. 2:**
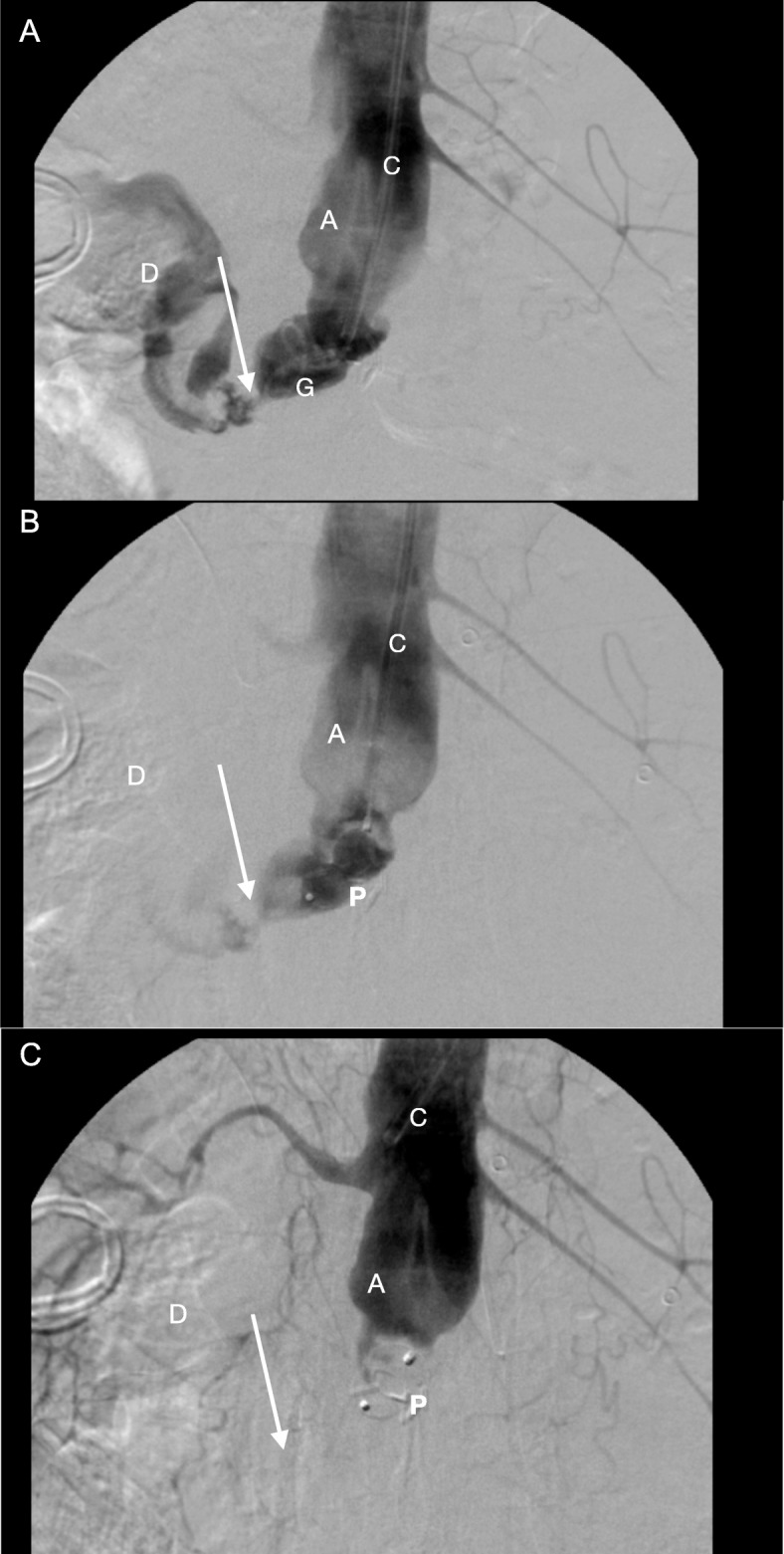
Angiography and the placement of the Amplatzer® Vascular Plug in the aortic stump. Angiography via the axillo-bi-femoral bypass demonstrating the infrarenal aortic stump (**a**), the duodenum (**d**) and the ADF marked with the arrow (↓). The angiographic catheter is marked with C and the guide wire with G. The upper graph (**a**) demonstrates contrast passage from the aorta through the ADF towards the duodenum. The middle graph (**b**) was taken 5 min following release of a 16-mm Amplatzer® Vascular Plug (P) within the aortic stump, and demonstrates minor contrast passage from the aortic stump towards the duodenal loop. The lower graph (**c**), 23 min later, demonstrates successful closure of the aortic stump with no further contrast leakage through the ADF

**Fig. 3:**
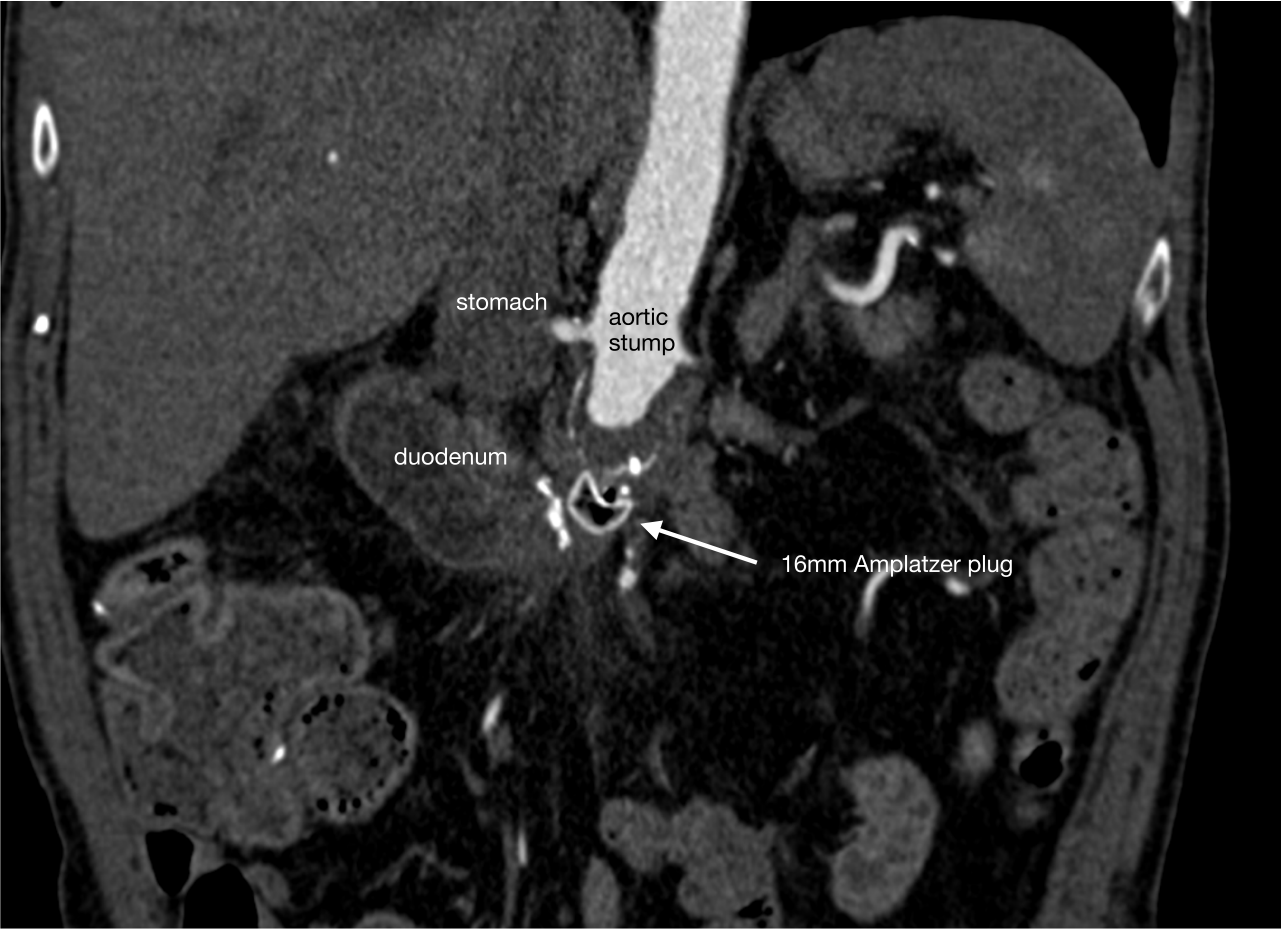
CTA abdomen with the Amplatzer® plug in situ after 3 months. CTA after 3 months showed thrombosis of the aortic stump with the Amplatzer® Vascular Plug in situ

Twelve weeks after surgery, the patient was still hospitalized and suddenly developed severe hematemesis with immediate hemodynamic instability. He was re-admitted to the Intensive Care Unit and was resuscitated and massively transfused with packed cells, fresh frozen plasma and thrombocytes. Gastroscopy showed large amounts of blood in the stomach but no active bleeding. The duodenum however, could not be reached. Computed tomography angiography (CTA) showed large amounts of blood in the duodenum and stomach, and extravasation of blood from the aortic stump into the duodenum (Fig. 1). Because of multiple prior abdominal operations, the patient was not considered suitable for open surgical repair. More important, he was in a hemorrhagic shock with a systolic blood pressure of 40 mmHg and therefore immediately transferred to the angiosuite. Percutaneous access was created by ultrasound-guided puncture of the axillo-bi-femoral bypass graft with introduction of an 8 mm sheath. Using a 4F multipurpose catheter within a 8F guiding catheter (Cardinal Health, Dublin, Ohio) we performed an angiography which confirmed the massive bleeding from the aortic stump towards the duodenal loop. A 16-mm AVP (Abbott Vascular, Santa Clara, California) was implanted within the aortic stump, which stabilized the patient within minutes. No other material or coils were used to cessate the bleeding. The second angiography performed 23 min after placement of the AVP confirmed that the bleeding had stopped (Fig. 2). Three months later, CTA demonstrated thrombosis of the aortic stump with the AVP in situ (Fig. 3). The patient was treated with intravenous antibiotics for half a year. He was discharged in a good clinical health to a rehabilitation clinic. After a follow-up of 4 years the patient was still alive.

## Discussion

The treatment for ADF consists of closure of the duodenal fistula, removal of the infected graft and vascular reconstruction using in situ repair or extra-anatomical bypass grafting (Batt et al. 2011; Bergqvist and Björck 2009; Burks et al. 2001). The mortality rates are high; 47–48% for in-situ repair and 33–51% for extra-anatomical repair (Deijen et al. 2016). Part of this mortality is caused by aortic stump ruptures or re-bleeding. The exact incidence of aortic stump ruptures is unknown.

Reviewing literature, O’Hara et al. analyzed the outcome of 84 infected abdominal aortic grafts of which 33 patients presented with an aortic *enteric* fistula (AEF). At a mean interval of 94 days following graft removal, aortic stump ruptures were the cause of death in 8 / 33 patients (24%) (O’Hara et al. 1986).

Kakkos et al. reviewed 823 patients with an AEF of which 62% were ADF. Open surgery was performed in 88% of the patients, extra-anatomical bypass grafting was done in 31% of the patients. In-hospital mortality was reported in 253 cases (31%). Postoperatively, a total of 23 patients died due to an aortic stump rupture; 12 within 30 days, and 11 after 30 days (Kakkos et al. 2016). Due to a limited follow-up and inhomogeneous group of patients, the exact incidence of stump ruptures in ADF could not be retrieved.

Classical treatment of an aortic stump rupture includes immediate re-laparotomy, removal of infected tissue, aortic stump over sewing and reinforcement with soft tissue flaps. However, re-laparotomy is almost never the most suitable solution since a more rapid response is often necessary. Also, within our case our patient nearly died due to a severe hemorrhagic shock. He entered the angiosuite with a systolic pressure of 40 mmHg. An endovascular treatment was the only solution to save the patient’s life.

Literature search revealed three cases in which endovascular treatment of aortic stump blow-out has been described (Marone et al. 2012; Wang et al. 2018; Terasaki et al. 1990).

Marone et al. reported a case of an aortic stump rupture nine years after treatment of an ADF. The stump rupture was treated endovascularly by brachial access using a 9F sheath and a 18-mm Amplatzer® plug (Abbott Vascular, Santa Clara, California). The plug successfully stopped the bleeding within 10 min. Five days after this procedure additional treatment was performed with removal of the plug, over sewing the aortic stump and performing duodenal repair. During laparotomy the distal end of the plug was found to be partially protruded through the aortic wall. The authors conclude that definitive surgical treatment, if possible, must be performed (Marone et al. 2012).

Wang et al. treated a persistent aortic stump bleeding trans-lumbarly using a 22-mm Amplatzer® plug (Abbott Vascular, Santa Clara, California). They placed additional coils during the procedure to obtain hemostasis. After the procedure the patient had several re-bleeds which were treated with additional coil placement. Despite this treatment, the patient expired 5 months postoperatively due to a re-bleed (Wang et al. 2018).

Finally, Terasaki et al. successfully performed transcatheter embolization of an aortic stump with 8-mm Gianturco® coils (Cook Medical, Bloomington, Indiana). In order to obtain hemostasis, additional gelatin-sponge pledgets were inserted within the aortic stump. The patient was still alive after a follow-up of 150 days postoperatively (Terasaki et al. 1990).

The choice of embolization device merits consideration. In the present case, but also in the cases described by others (Marone et al. 2012; Wang et al. 2018), an Amplatzer plug was used. This plug needs a larger access (8/9F), but has the advantage of a more rapid procedure with faster cessation of bleeding and less risk of migration. Finally, the plug has a longer delivery wire (135 cm), making it suitable to be delivered from a brachial or an axillary access point. The use of coils has the advantage of a smaller access, but has the disadvantage of a longer procedure with multiple steps. Furthermore, additional coil placement or gelatin-sponge pledgets are frequently necessary to create hemostasis. Finally, there is a risk of migration of the coils towards the fistula or renal and mesenteric artery through reflux. For both the coils and the Amplatzer® plug it is important to realize that the devices work through clot formation and therefore do not immediately stop the bleeding. A proper coagulation is therefore of great importance. Within our case the AVP was deliberately released within the aortic stump and not inside the fistula. We think that placement of the plug within the chronic infected aortic wall has a risk of further protrusion throughout the aortic wall with additional risk of re-bleed. Finally, the long-term implications of this endovascular approach remains to be elucidated. Marone et al. used this treatment as a bridge to surgery while we chose to treat our patient conservatively. ADF and especially aortic stump rupture treatment is a rare entity and therefore limited evidence is available. The decision to use this endovascular treatment as a bridge to surgery or as a definitive treatment should be based on the patient’s anatomy and clinical condition.

## Conclusions

Rapid treatment is required in case of an aortic stump rupture. Endovascular treatment might be a suitable option. Whether this endovascular treatment of an aortic stump rupture is a definitive treatment or a bridge to further surgery remains to be elucidated.

## Data Availability

Not applicable.

## Abbreviations

AAA: Abdominal aortic aneurysm
ADF: Aortoduodenal fistula
AEF: Aortic enteric fistula
AVP: Amplatzer® vascular plug
CTA: Computed tomography angiography
